# Cross-sectional seroprevalence study of measles antibodies among children to identify gaps in population immunity, Ireland, 2024

**DOI:** 10.2807/1560-7917.ES.2026.31.9.2500313

**Published:** 2026-03-05

**Authors:** Melissa Brady, Lieke Brouwer, Katie O’ Brien, Michael Carton, Laura Whitton, Claire Dillon, Jane Finucane, Fiona Culkin, Ellen Perry, Elaine Brabazon, Sarah Gee, Kate Browne, Deirdre Burke, Cillian De Gascun, Ann Leonard, Robert Cunney, Lois O’ Connor

**Affiliations:** 1 Health Service Executive Health Protection Surveillance Centre (HPSC), Dublin, Ireland; 2 Public Health Laboratory, Cherry Orchard Hospital, Health Service Executive (HSE), Dublin, Ireland; 3 European Programme for Public Health Microbiology Training (EUPHEM), European Centre for Disease Prevention and Control, (ECDC), Stockholm, Sweden; 4 UCD National Virus Reference Laboratory, Dublin, Ireland; 5 Tallaght University Hospital, Dublin, Ireland; 6 Children’s Health Ireland at Temple Street, Dublin, Ireland; 7 Department of Microbiology, Royal College of Surgeons in Ireland (RCSI), Dublin, Ireland

**Keywords:** Measles, immunity, vaccine, IgG antibody, seroprevalence, surveillance, Ireland

## Abstract

**BACKGROUND:**

Global resurgence of measles highlights the need for countries to identify and address immunity gaps. This is challenging given antibody waning and, in Ireland, the absence of robust national vaccine coverage data, population changes due to migration and outdated population-level seroprevalence data from the last national serosurvey (2003).

**AIM:**

We aimed to determine the seroprevalence of measles IgG in children aged 3–17 years in Ireland.

**METHODS:**

Convenience sampling of anonymised residual serum samples from four hospital laboratories across four of six health regions was conducted between 1 February and 19 June 2024. Samples were tested for measles IgG antibodies using a commercial chemiluminescence immunoassay. Seropositivity was adjusted for test sensitivity and specificity and was calculated by sex, age and location.

**RESULTS:**

In total 2,509 of 2,924 samples were seropositive and 415 were seronegative indicating measles IgG seroprevalence of 90.3% (95% confidence interval (CI): 89.2–91.4), with no significant difference by sex. Children aged 3–5 years (94.9%; 95% CI: 92.4–96.6) and 6–9 years (94.2%; 95% CI: 91.7–95.9) had significantly higher seropositivity when compared with children aged 10–13 years (89.1%; 95% CI: 86.6–91.3) and 14–17 years (87.6%; 95% CI: 85.5–89.4).

**CONCLUSION:**

Our findings suggest close to adequate protection against measles among children 3–9 years but suboptimal (< 95%) protection among children aged 10–17 years. This immunity gap is not reflected in measles vaccine coverage data, highlighting the utility of seroprevalence data to enhance knowledge of clinical protection at population level and to inform vaccination strategies.

**Key public health message**

**What did you want to address in this study and why?**

Due to the increase in measles in recent years, countries need to identify and address immunity gaps. Measles vaccination coverage data alone cannot reliably determine immunity in Ireland due to vaccination doses 1 and 2 records stored in separate systems, natural antibody waning in vaccinated people, and population movement. We studied measles IgG seroprevalence to identify measles immunity gaps among children in Ireland.

**What have we learnt from this study?**

While measles IgG antibody seroprevalence among 3–9-year-olds was near adequate, in 10–17-year-olds it was lower than the ≥ 95% target rate of vaccination coverage required to eliminate measles. The findings indicate potential susceptibility to infection among older children. This is not evident in national vaccination coverage data, highlighting the use of seroprevalence data to enhance knowledge of population immunity.

**What are the implications of your findings for public health?**

Measles seroprevalence studies complement vaccine coverage data. Our findings were used in a national catch-up vaccination programme for measles, and we recommend that this study be repeated periodically to meet public health needs in Ireland.

## Introduction

Measles is among the world’s most contagious diseases, infecting the respiratory tract and causing systemic symptoms. Immunocompromised people, unvaccinated pregnant women and unvaccinated young children are at highest risk of severe disease, complications and death [1,2]. Community-wide immunisation is the most effective preventive measure, with an estimated 57 million deaths worldwide averted between 2000 and 2022 due to the vaccine [3]. Despite many countries having achieved measles elimination status, including Ireland, the World Health Organization (WHO) reported in 2019 that a global resurgence of measles posed a risk to undermining key gains made in controlling the virus [4,5]. To achieve and maintain measles elimination (defined as the interruption of endemic measles transmission in a defined geographic area for at least 12 months in the presence of a well-performing surveillance system), a high level (≥ 95%) of population immunity is required. Yet global vaccination rates in the 10 years up to 2019, were ca 10% below the recommended threshold for the first dose and 25% below for the second dose [4,6].

Factors influencing suboptimal vaccine uptake include low levels of vaccine acceptance and inequitable vaccine access. For example, the decline in vaccination uptake in the 1990s and early 2000s in England and elsewhere followed media coverage of the since retracted Wakefield study falsely linking measles-mumps-rubella (MMR) vaccination to autism [7]. In addition, the COVID-19 pandemic caused setbacks worldwide due to the suspension of immunisation services [3]. By the end of 2023, none of the six WHO Regions successfully achieved and sustained measles elimination [8]. In the United Kingdom (UK) and in several other European countries, a recent upwards trend in cases and outbreaks is evident [9,10]. The European Centre for Disease Prevention and Control (ECDC) highlighted concerns around suboptimal vaccine coverage in 2024. In their measles threat assessment they classified the risk as moderate among infants, unvaccinated children and immunocompromised people, with a high probability of importation of measles between European Union (EU) countries and exportation outside the EU/European Economic Area (EEA) due to movement of people [10].

In 2024, 35,212 measles cases were reported in the EU/EEA, a 10-fold increase on 2023, with highest notification rates in Romania, Austria, Belgium and Ireland. Among cases with known vaccination status, 87% were unvaccinated. Twenty-three deaths were attributed to measles, 22 in Romania and one in Ireland [11]. Of the 208 (4.0/100,000 population) cases notified in Ireland in 2024, the majority (n = 139; 67%) were among people aged 18 years or younger, and among cases with known vaccination status, most (n = 104; 80%) were unvaccinated (data not shown).

**Figure 1:**
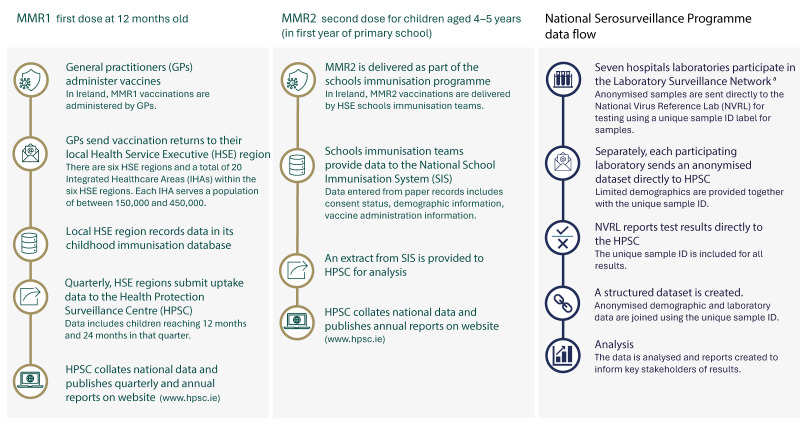
Process flows for national measles-mumps-rubella vaccine uptake reporting and National Serosurveillance Programme workflow, Ireland

GP: general practitioner; HPSC: Health Protection Surveillance Centre; HSE: Health Service Executive; ID: identification; IHA: integrated healthcare area; LSN: Laboratory Surveillance Network; MMR1: measles-mumps-rubella vaccine first dose; MMR2: measles-mumps-rubella vaccine second dose; NVRL: National Virus Reference Laboratory; SIS: National School Immunisation System.

^a^ The LSN was initially set up on a voluntary basis in 2021 to provide anonymised samples for COVID-19 serosurveillance. Since 2022, the programme has expanded to include serosurveillance of other notifiable diseases such as measles. For the paediatric measles serosurveillance project, 4 of the 7 LSN partners were able to participate.

To determine population immunity, WHO advises that documenting immunity for each cohort born since the introduction of the vaccine in the national immunisation programme is sufficient, as most people born before this date have naturally acquired immunity [6]. However, robust and reliable information on immunity or vaccine coverage is not always available at national level. Measles is a notifiable disease in Ireland since 1948. A nationwide measles vaccine was first introduced in 1985, which was replaced with a single-dose MMR vaccine in 1988 and a two-dose regimen in 1992 [12]. The first dose (MMR1) is offered at 12 months of age in primary care, and the second dose (MMR2) is offered between 4 and 5 years of age mostly through the schools immunisation programme [13] (Figure 1). Measles vaccine uptake varies by year but is consistently below the WHO 95% threshold. Gaps in our knowledge of vaccine coverage in Ireland exist because records are unlinked and reflect the uptake of either MMR1 or the uptake of MMR2 but not both doses. Furthermore, these records relate to the national immunisation programme and do not include vaccine catch-up programmes [14]. Moreover, they do not account for rapid changes in the population due to population movement. In 2022, the proportion of people who usually lived in Ireland but were born elsewhere stood at 20% (1,017,437 people) of the population, an increase of 3% (207,031 people) from 2016. The highest proportion of non-Irish born residents in 2022 originated from Brazil, India, Romania and Ukraine [15].

Seroprevalence studies can provide insights into population immunity. Ireland’s National Seroprevalence Programme (NSP) is a collaboration between the Health Service Executive (HSE) Health Protection Surveillance Centre (HPSC), National Virus Reference Laboratory (NVRL) and the Laboratory Surveillance Network (LSN), a network of seven primary diagnostic laboratories across Ireland (Figure 1). While these data are outdated, the latest measles seroprevalence data for children in Ireland (from the European Sero-Epidemiology Network 2 study in 2003) showed Ireland was a country with higher levels of susceptibility than the WHO targets recommend, particularly in younger age groups (aged 2–9 years) [16]. Data from a study among adults in Ireland in 2022 showed higher seronegativity among younger adults (aged 18–19 years), again highlighting potential susceptibility among adjacent younger age groups [17]. We prospectively investigated measles seroprevalence among children aged 3–17 years in Ireland to identify immunity gaps and to inform the MMR catch-up programme established by the National Incident Management Team (NIMT) for measles, formed in 2024 as part of the Irish response to the measles threat.

## Methods

### Study initiation

This study was initiated by the HSE HPSC and was undertaken by the NSP. Measles serosurveillance was prioritised in 2022 following stakeholder and NSP Steering Committee engagement. Although initiated in October 2022, commencement of this study was delayed until 2024, mainly due to difficulties recruiting dedicated hospital sample management personnel during a time of substantial recruitment challenges in the medical laboratories of Ireland’s hospitals [18]. The measles NIMT endorsement for the project accelerated study commencement.

### Study population, setting and sampling methods

Convenience sampling of anonymised residual serum samples, left over after diagnostic testing, was conducted by the LSN in the clinical laboratories of four large public hospitals between 1 February 2024 and 19 June 2024. The hospitals were geographically distributed across four of the six HSE health regions in Ireland: St. Vincent’s University Hospital in the Dublin and South East region, Tallaght University Hospital in the Dublin and Midlands region, University Hospital Limerick in the Mid West region, and Letterkenny University Hospital in the West and North West region. The hospital catchment areas for the four hospitals covered a total of 22% (n = 1,156,532) of the population based on the 2022 census of Ireland [19].

Samples were collected from children [20] aged 3 to 17 years, from sources that best represented the general population (primary care, emergency departments, outpatient departments, urgent care centres), and excluded hospital inpatients. Children younger than 3 years of age were excluded as it was difficult to obtain serum samples in sufficient quantities for this group.

A minimum sample size of 388 per age group (3–5yrs, 6–9yrs, 10–13yrs, 14–17yrs, n = 1,552 in total) was calculated using the Epi R package, R statistical software version 4.2.2 [21], based on an estimated measles IgG seroprevalence of 88%, a precision of 4%, assay sensitivity of 94.7% and assay specificity of 97.4%. In total, 2,924 samples were included due to unintended oversampling of older children and continued sampling to obtain sufficient sample numbers for younger children. Sample size targets in all age groups were exceeded. The number of samples provided by each laboratory location was based on capacity at that location.

### Laboratory testing

All laboratory testing was conducted by the NVRL using the LIAISON XL Measles IgG qualitative chemiluminescent immunoassay (DiaSorin, Saluggia, Italy). Samples were reported as either seronegative (< 13.5 AU/mL) or seropositive (≥ 16.5 AU/mL). Those with equivocal results (13.5–16.4 AU/mL) were retested and if still equivocal were classified as seropositive. The cut-off value discriminating between the presence and absence of measles virus IgG is 15.0 AU/mL, which equates to 175 mIU/mL WHO Third International Standard for Anti-Measles [22].

### Data sources and collection

A complete, minimum dataset (date of birth, sex, county of residence, sample collection date, sample source, laboratory location and IgG test result) was collected by standard template using Microsoft Excel software. National level data on MMR1 uptake at 24 months and on the incidence of measles in Ireland were sourced from the HPSC (data not shown) [23,24]. Data on MMR uptake at primary school age (MMR2) were not used due to uncertainties in the dataset.

### Statistical analysis

Analysis was performed using R statistical software version 4.2.2. Seroprevalence was calculated overall and by age, sex (registered as a binary variable), laboratory location and sample source, adjusting for diagnostic misclassification using the Rogan-Gladen method [25]. Results were weighted to the national population structure by age and sex. The main results presented were adjusted but non-weighted.

Median quantitative antibody concentrations and interquartile ranges (IQR) (within qualitative range limitations of 5–300 AU/ml) were calculated by age group and compared using Student’s t-test.

We performed univariate and multivariable logistic regression (MVA) to explore independent predictors (sex, age, county of residence, laboratory location, sample source) of measles seronegativity using chi-square and likelihood ratio tests. We used forward stepwise techniques and excluded variables based on model efficiency, according to the Akaike Information Criterion. Collinearity was explored using variance inflation factor. Potential confounders and effect modifiers (age, sex) were explored in stratified analyses. Estimates (including 95% confidence interval (CI)) of association of independent variables with seronegativity were calculated and presented for best overall multivariable model. A statistical significance level of p < 0.05 was used.

## Results

### Demographic data

Of 2,924 samples, 1,346 (46%) were from males, median age was 12 years (IQR: 7–15 years). Samples were collected from children residing in 19 of 26 counties in Ireland, nearly half (49%; n = 1,443) resided in county Dublin (Table 1).

**Table 1:** Demographic characteristics, measles seroprevalence study, Ireland, 1 February 2024–19 June 2024 (n = 2,924 samples)

Characteristics	n	%
Sex		
Male	1,346	46.0
Female	1,578	54.0
Age group (years)		
3–5	480	16.4
6–9	524	17.9
10–13	732	25.0
14–17	1,188	40.6
Sex and age group (years)		
Male 3–5	249	8.5
Male 6–9	275	9.4
Male 10–13	329	11.3
Male 14–17	493	16.9
Female 3–5	231	7.9
Female 6–9	249	8.5
Female 10–13	403	13.8
Female 14–17	695	23.8
Laboratory location		
St. Vincent’s University Hospital	726	24.8
Tallaght University Hospital	1,357	46.4
University Hospital Limerick	162	5.5
Letterkenny University Hospital	679	23.2
County of residence		
Dublin	1,443	49.4
Donegal	680	23.3
Wicklow	355	12.1
Kildare	229	7.8
Limerick	107	3.7
Other	110	3.8
Sample source		
Emergency department	759	26.0
General practitioner	1,563	53.5
Outpatient department	439	15.0
Urgent care centre	163	5.6

### Seropositivity

In total, 2,509 samples (85.8%; 95% CI: 84.5–87.0) were seropositive (42 of which were equivocal) and 415 were seronegative. When weighted to the national population structure by age and sex, seropositivity was 86.4% (95% CI: 85.0–87.6). The adjusted (unweighted) seropositivity was 90.3% (95% CI: 89.2–91.4) (Table 2). The adjusted seropositivity did not significantly differ by sex. However, children aged 3–5 years (94.9%; 95% CI: 92.4–96.6) and 6–9 years (94.2%; 95% CI: 91.7–95.9) had significantly higher seropositivity when compared with children aged 10–13 years (89.1%; 95% CI: 86.6–91.3) and 14–17 years (87.6%; 95% CI: 85.5–89.4). Seropositivity varied by laboratory location, with the lowest seropositivity among those tested from the laboratory of St. Vincent’s University Hospital in Dublin. Conversely, by county of residence, seropositivity was lowest in Donegal (86.8%; 95% CI: 83.9–89.2) and Wicklow (88.0%; 95% CI: 84.1–91.1).

**Table 2:** Seropositivity by age, sex and location, measles seroprevalence study, Ireland, 1 February 2024–19 June 2024 (n = 2,924 samples)

Characteristics	n	n seropositive	Unadjusted % seropositivity	Adjusted % seropositivity^a^	95% CI
Total	2,924	2,509	85.8	90.3	89.2–91.4
Sex					
Male	1,346	1,144	85.0	89.5	87.7–91.0
Female	1,578	1,365	86.5	91.1	89.6–92.4
Age group (years)					
3–5	480	432	90.0	94.9	92.4–96.6
6–9	524	468	89.3	94.2	91.7–95.9
10–13	732	620	84.7	89.1	86.6–91.3
14–17	1,188	989	83.2	87.6	85.5–89.4
Sex and age group (years)					
Male 3–5	249	220	88.4	93.1	89.0–95.8
Male 6–9	275	246	89.5	94.3	90.7–96.6
Male 10–13	329	268	81.5	85.6	81.3–89.1
Male 14–17	493	410	83.2	87.5	84.1–90.2
Female 3–5	231	212	91.8	96.8	93.4–98.6
Female 6–9	249	222	89.2	94.0	90.1–96.5
Female 10–13	403	352	87.3	92.0	88.8–94.4
Female 14–17	695	579	83.3	87.6	84.9–89.9
Laboratory location					
Letterkenny University Hospital	679	560	82.5	86.7	83.9–89.1
University Hospital Limerick	162	144	88.9	93.7	88.5–96.7
St. Vincent’s University Hospital	726	579	79.8	83.8	80.8–86.3
Tallaght University Hospital	1,357	1,226	90.3	95.3	94.0–96.3
County of residence					
Dublin	1,443	1,250	86.6	91.2	89.6–92.6
Donegal	680	561	82.5	86.8	83.9–89.2
Wicklow	355	297	83.7	88.0	84.1–91.1
Kildare	229	207	90.4	95.3	91.5–97.5
Limerick	107	96	89.7	94.6	88.0–97.8
Other	110	98	89.1	93.9	87.2–97.4
Sample source					
Emergency department	759	656	86.4	91.0	88.7–92.9
General practitioner	1,563	1,319	84.4	88.8	87.1–90.3
Outpatient department	439	406	92.5	97.6	95.5–98.7
Urgent care centre	163	128	78.5	82.4	75.5–87.8

CI: confidence interval.

^a ^Adjusted for diagnostic misclassification.

### Quantitative antibody results

**Figure 2:**
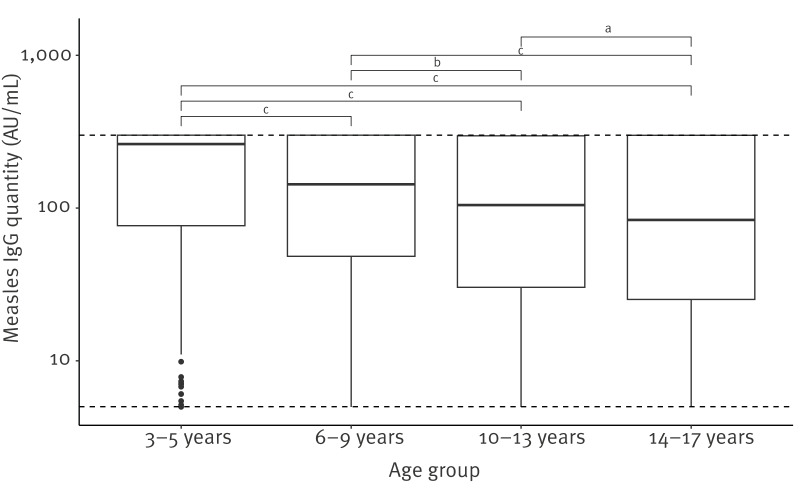
Median quantitative antibody ranges by age group, measles seroprevalence study, Ireland, 1 February 2024–19 June 2024 (n = 2,294 samples)

^a^ No significant difference between age groups (p > 0.05)

^b^ Significant difference p ≤ 0.001

^c^ Significant difference p ≤ 0.0001

The median quantitative antibody titre was 119.0 AU/mL (IQR: 34.2–300.0). In line with the results of the qualitative analysis, quantitative antibody titre was lower for older children (Figure 2).

The boxplot shows the median, interquartile ranges, minimum–maximum ranges and outliers. Dashed lines represent the upper and lower detection limits of the laboratory assay.

### Results of multivariable analysis

Variables in the final MVA model were age, sex, sample source and county of residence (Table 3). The odds of seronegativity were significantly (p < 0.001) higher for children aged 10–13 years (odds ratio (OR): 1.48; 95% CI: 1.0–2.2) and 14–17 years (OR: 1.61; 95% CI: 1.1–2.3) when compared with younger children. Sex alone did not differ significantly in the MVA, but when age group and sex were combined in the univariate analysis, males aged 10–13 years had significantly higher odds of seronegativity compared to other sex and age group combinations (OR: 1.73; 95% CI: 1.1–2.8). Children residing in one county (Donegal) had higher seronegativity when compared with children residing elsewhere (OR: 1.74; 95% CI: 1.1–2.9), and those whose samples originated from outpatient departments had lower seronegativity when compared with other sample sources (OR: 0.55; 95% CI: 0.4–0.8).

**Table 3:** Univariate and multivariable analysis of factors associated with seronegativity, measles seroprevalence study, Ireland, 1 February 2024–19 June 2024 (n = 2,924 samples)

Category	n	n seropositive	Univariate analysis				Multivariable analysis			
			OR	95% CI lower	95% CI upper	p value^a^	OR	95% CI lower	95% CI upper	p value^a^
Sex										
Female	1,578	1,365	Ref			0.2442	Ref			0.069
Male	1,346	1,144	1.13	0.92	1.39		1.22	0.99	1.50	
Age (years)	2,924	2,509	***1.06***	***1.03***	***1.09***	* * ***< 0.001***	NI			
Age group (years)										
3–5	480	432	Ref			* * ***< 0.001***	Ref			* * *** 0.005***
6–9	524	468	1.08	0.72	1.62		1.02	0.68	1.54	
10–13	732	620	***1.63***	***1.14***	***2.35***		***1.48***	***1.03***	***2.16***	
14–17	1,188	989	***1.81***	***1.31***	***2.56***		***1.61***	***1.14***	***2.30***	
Sex and age group (years)										
Male 3–5	249	220	Ref			* * ***< 0.001***	NI			
Male 6–9	275	246	0.89	0.52	1.55					
Male 10–13	329	268	***1.73***	***1.08***	***2.81***					
Male 14–17	493	410	1.54	0.99	2.45					
Female 3–5	231	212	0.68	0.37	1.24					
Female 6–9	249	222	0.92	0.53	1.61					
Female 10–13	403	352	1.10	0.68	1.80					
Female 14–17	695	579	1.52	1.00	2.39					
Laboratory location										
Tallaght University Hospital	679	560	Ref			* * ***< 0.001***	NI			
Letterkenny University Hospital	162	144	***1.99***	***1.52***	***2.60***					
St. Vincent’s University Hospital	726	579	***2.38***	***1.84***	***3.07***					
University Hospital Limerick	1,357	1,226	1.17	0.67	1.92					
County of residence										
Kildare	229	207	Ref			* * ***< 0.0339***	Ref			* * ***< 0.043***
Dublin	1,443	1,250	1.45	0.93	2.37		1.22	0.77	2.00	
Wicklow	355	297	***1.84***	***1.11***	***3.15***		1.50	0.90	2.60	
Limerick	107	96	1.08	0.49	2.27		0.99	0.44	2.11	
Donegal	680	561	***2.00***	***1.26***	***3.31***		***1.74***	***1.09***	***2.90***	
Other	110	98	1.15	0.53	2.39		1.10	0.50	2.30	
Sample source										
Emergency department	759	656	Ref			* * ***< 0.001***	Ref			* * ***< 0.001***
General practitioner	1,563	1,319	1.18	0.92	1.52		1.12	0.87	1.46	
Outpatient department	439	406	***0.52***	***0.34***	***0.77***		***0.55***	***0.36***	***0.82***	
Urgent care centre	163	128	1.74	1.12	2.65		1.57	0.99	2.45	

CI: confidence interval; NI: not included in final model due to collinearity; Ref: reference category.

^a^ By chi-square test and likelihood ratio test.

Significant values p < 0.05 are highlighted in bold italics.

### Comparison of seropositivity with first dose measles-mumps-rubella vaccination uptake and measles incidence

Between 2008 and 2023, MMR1 uptake at 24 months of age (birth cohort 2006–2021) was relatively stable in Ireland with an average uptake of 91.3%, ranging from lowest of 88.7% in 2008 to highest of 93.0% in 2014, but consistently below the WHO 95% target. While MMR1 uptake and seropositivity were both relatively steady during the study period, MMR1 uptake was on average 3.5% higher than the adjusted measles IgG seroprevalence for those born between 2006 and 2012, and 3.1% lower than the seroprevalence for those born between 2013 and 2021 (Table 4).

**Table 4:** Seropositivity by birth cohort and first dose measles-mumps-rubella vaccine uptake at 24 months for the same period, measles seroprevalence study, Ireland, 1 February 2024–19 June 2024 (n = 2,294 samples)

Calendar year	Vaccination coverage at 24 months of age % (year of birth for study participants)	National annual measles cases/100,000 population	n study participants	n seropositive	Adjusted seropositivity %	95% CI
2008	88.7 (2006)	1.30	95	80	88.6	80.0–93.9
2009	90.1 (2007)	3.50	275	230	88.0	83.4–91.5
2010	90.1 (2008)	8.80	361	292	85.0	80.8–88.4
2011	91.9 (2009)	5.80	385	321	87.7	83.9–90.7
2012	92.4 (2010)	2.20	283	239	88.9	84.5–92.2
2013	92.6 (2011)	1.10	186	158	89.4	83.9–93.3
2014	93 (2012)	0.70	162	134	87.0	80.6–91.6
2015	92.9 (2013)	0.04	135	121	94.5	88.8–97.5
2016	92.5 (2014)	0.90	139	124	94.0	88.3–97.2
2017	92.2 (2015)	0.50	126	111	92.8	86.5–96.5
2018	92.3 (2016)	1.60	151	135	94.2	88.9–97.2
2019	91.3 (2017)	1.30	120	108	94.9	88.8–97.9
2020	91.8 (2018)	0.10	134	125	98.5	94.1–99.7
2021	90.4 (2019)	0.00	157	140	94.0	88.7–97.0
2022	89.5 (2020)	0.04	177	157	93.5	88.5–96.5
2023	89.5 (2021)	0.08	38	34	94.3	80.4–98.9

CI: confidence interval.

## Discussion

The overall seroprevalence of measles IgG antibodies (90.3%) was lower than 95%, the WHO target rate of vaccine coverage to eliminate measles.

Seropositivity among children aged 3–5 years and 6–9 years was relatively high (94.9% and 94.2%, respectively). This is a reassuring and welcome finding given the increase in cases and outbreaks in Ireland and globally in recent years. For those born between 2013 and 2021, the seroprevalence suggests higher protection against measles than is evident in MMR1 uptake data, possibly due to high uptake of two MMR doses (despite a slight decline in MMR2 coverage during the COVID-19 pandemic in the years 2021 and 2022) and vaccines administered through catch-up campaigns [26]. It is possible, however, that MMR1 uptake data may be inaccurate. Reasons for this include: challenges related to denominator accuracy due to movement of children within and outside the jurisdiction, inaccurate vaccine uptake data for children who receive vaccines at an age older than that recommended by national programmes and variable record keeping practices. These challenges highlight the importance of augmenting national vaccine uptake data with national serosurveillance, as recommended by WHO, to provide an accurate picture of population immunity [27].

Lower seropositivity among those aged 10–13 years and 14–17 years (89.1% and 87.6% respectively), which was significant in the MVA, indicates potential susceptibility to infection among a cohort of older children. This is not evident in vaccination coverage data, in which the MMR1 uptake at 24 months is higher than the seropositivity for children born between 2006 and 2012. This is contrary to expectations that seropositivity would likely be higher than MMR1 uptake due to added protection from MMR2, vaccine catch up campaigns and immunity acquired through natural infection (although this is expected to be low for children in Ireland). Natural waning of antibodies among vaccinated children who have not had subsequent natural infection or exposure to the virus may in part explain this deficit. In a study of measles antibody decline among adults in Italy in 2020, neutralising antibody titres inversely correlated with the time elapsed since the second vaccine dose, particularly over 12–14 years [28]. A similar result was found for the adult and paediatric population in Germany [29,30]. In Belgium this decline was noted for some birth cohorts but not others, likely due to a combination of antibody waning, exposure to natural infection and catch-up vaccination among different birth cohorts [31]. In a 2022 study of measles antibody seroprevalence among adults aged 18–34 in Ireland, seronegativity was highest (14.7%) for the youngest adults aged 18 and 19 years (2003–2004 birth cohort) [17]. These findings, along with the findings of our study provide evidence of a decline in seropositivity at ca 10–15 years post-vaccination. However, multiple factors, including migration and variations in reporting of vaccine coverage, may make findings difficult to interpret. It is also possible that our study may not be nationally representative when compared with national MMR1 coverage.

While no difference was observed by sex alone, males aged 10–13 years had significantly lower seropositivity (85.6%) than other sex/age group combinations. This enhances our knowledge, particularly as MMR uptake data are not available by sex. Geographic location (county of residence) within Ireland was also significant in the MVA. Those residing in counties Wicklow and Donegal, which are situated in the east coast and north west coast of Ireland, respectively, had lower seropositivity, although this was not significant for Wicklow in the MVA. Reasons for geographic differences are not clear, although vaccination uptake/delivery/access, deprivation, population movements and other demographic factors or clinical characteristics may play a role. Geographical differences in vaccine access/delivery include that MMR2 is delivered to children through GPs in Donegal, whereas it is delivered through immunisation teams in school settings in most other counties, which may improve uptake due to more convenient access. Deprivation also differs geographically. Donegal is situated in the catchment area of Letterkenny University Hospital where a considerably higher proportion (36.2%) of disadvantaged people reside when compared with the national average of 22.0% [19]. Region of residence and migration history were associated with measles seronegativity in a German study [29], but migration history was not available in our dataset. In addition, those whose samples originated from outpatient departments had higher seropositivity. It is possible that children who attend outpatient departments may have underlying conditions and medical vulnerabilities that result in a higher likelihood of vaccine uptake, or the administration of exogenous antibody. Unfortunately, information on the medical specialties from which samples were received was not collected. However, as it would enhance our understanding of the data, we recommend that this be collected in future studies.

The target rate of vaccination coverage to eliminate measles assumes that all vaccinated persons develop protective immunity. Commercial IgG immunoassays such as the LIAISON assay used in this study can be useful to obtain rapid results, but measles IgG titres generally do not indicate clinical protection status, for which neutralising antibody activity is more appropriate. However, measuring neutralising antibody activity is prohibitively expensive and labour intensive for large observational studies of this nature. The LIAISON assay may be less effective at detecting measles IgG post vaccination when compared with measles IgG post infection, as titres are often lower and decline more rapidly. That being said, correlation between IgG titres obtained using the LIAISON assay and neutralising antibody titres has been demonstrated by researchers for all tested measles virus genotypes (Edmonston, B3, C2, D4, D8 and H1), indicating that it can potentially be a predictor of clinical protection [32]. However, the reported correlation was weak, and inferior to that of a comparator assay [32]. In our study, IgG titres from the LIAISON assay were not correlated with neutralising antibody titres. Nonetheless, measles IgG seroprevalence accounts for humoral immunity only and may underestimate total protective immunity. Cellular immunity might be present and re-stimulated in a person to boost IgG antibodies on second exposure to the same antigen, although the proportion of individuals who might experience this anamnestic response is not known [28].

This study was used to reaffirm the inclusion of children aged 17 years and under in target group prioritisation in a national measles vaccine catch up campaign which commenced in March 2024. The data will also be used in future catch-up campaigns. A new digitalised National Immunisation Information System for Ireland, currently in development, aims to address gaps in vaccination uptake data (e.g. linkage of vaccine records, inclusion of ethnicity and country of birth information), although seroprevalence data will be of continued value given changes in population immunity due to antibody waning and population movement. To maintain measles elimination and a high level of immunity, targeted vaccination strategies and good quality surveillance including population seroprevalence studies are needed. We propose that measles seroprevalence should be determined 10–15 years after last vaccination and more frequently in countries of considerable migration or following e.g. pandemic disruptions. Studies to evaluate the success of vaccine catch-up campaigns may also be useful. Our data may also be used by other European countries with similar disease epidemiology to assess their vaccination strategies.

Although our study had several strengths, including a large sample size and a complete minimum dataset, there were limitations. We used convenience sampling methods which are readily accessible but more restrictive and considerably less resource intensive than probability sampling techniques. This was done because variables of interest that could help to further tailor vaccination campaigns and public health messaging (vaccination history, infection history, country of birth, ethnicity) are often unavailable. We conducted age and sex weighting to improve representativeness and minimise selection bias, but weighting by geographic location was not possible as not all locations were included. Geographic representativeness was reasonable, with samples from hospital laboratories in four of six health regions in Ireland, but including samples from all six regions would be preferable. Therefore, geographic differences should be interpreted with caution. Although we could not include information on country of birth or ethnicity, we tried to ensure inclusion of migrant and ethnic minority groups by incorporating a range of sample sources since people who have migrated to Ireland may not yet be linked in with primary care due to high service demands. We acknowledge that age-specific susceptibility targets could be helpful given that very young children would not be expected to meet the 95% threshold. It is estimated that 95% of children respond to the first vaccine dose, whereas 99% are protected after two doses [7]. This is reflected in age-specific targets used in the UK, with lower targets of 85% for those aged between 1 and 4 years [7]. Generating age-specific targets is complicated and, encompasses several factors including vaccination coverage, pre-school mixing patterns, school starting age, population demographics and risks of imported cases. As such, good public health rationale is needed to invest resources into developing these targets for Ireland.

## Conclusion

While measles IgG antibody seroprevalence among younger children of 3–9 years in Ireland is sufficient (>94%), we found that in older children it was lower than the WHO target rate of 95% vaccination coverage required to eliminate measles. We believe that this study demonstrates the value of serosurveillance to enhance knowledge of clinical protection against measles at the population level, and to inform vaccination strategies, particularly in countries of high vaccine coverage and population movement. Serosurveillance complements vaccine coverage data, and we recommend that it be conducted periodically in Ireland to identify immunity gaps and inform interventions to improve vaccine coverage.

## Data Availability

All data presented in the manuscript are available from the corresponding author upon request.
